# Role of Surgery in Brucella Spondylodiscitis: An Evaluation of 28 Patients

**DOI:** 10.7759/cureus.33542

**Published:** 2023-01-09

**Authors:** İlker Deniz Cingöz

**Affiliations:** 1 Department of Neurosurgery, Faculty of Medicine, Usak University, Usak, TUR

**Keywords:** surgery, spondylodiscitis, posterior instrumentation, lumbosacral brucellosis, interbody fusion

## Abstract

Background

A limited number of studies are available in the literature on the surgical treatment of brucellosis-related spondylodiscitis. This study aimed to define and discuss the role of surgery in brucellosis-related spondylodiscitis.

Methodology

A total of 28 patients who underwent surgical treatment due to brucellosis-related spondylodiscitis between February 2021 and August 2022 were included in this study. Medical records, radiological images, and laboratory data were collected. Surgical results were evaluated according to the Visual Analog Scale (VAS) and Oswestry Disability Index (ODI). C-reactive protein (CRP) and erythrocyte sedimentation rate (ESR) levels, clinical findings, postoperative radiological imaging findings, and complications were evaluated.

Results

In total, 16 of the patients included in the study were male and 12 were female, with a mean age of 56.4 ± 7.2 years. The mean follow-up duration was 11.8 ± 5.4 months. Brucellosis involvement was in the lumbosacral region in all patients. Overall, 21 patients had a neurological deficit in the preoperative period. Posterior stabilization and fusion were done in 20 (71.4%) patients, while simple laminectomy (decompression) and debridement were done in eight (28.6%) patients. There was a decrease in the pain in the lower back and leg in all patients in the postoperative period. Neurological recovery was achieved in 18 patients with a neurological deficit. Two patients underwent wound drainage in the postoperative period. The patient who had morbid obesity and comorbidities died in the postoperative period. ESR and CRP levels returned to normal at the end of the six-month follow-up. There was a significant recovery in VAS and ODI scores (p < 0.05). In total, 24 (85.4%) patients were considered fully recovered both radiologically and clinically at the end of the follow-up.

Conclusions

Although long-term and specific antibiotic treatment constitute the main treatment in brucellosis-related spondylodiscitis, debridement, decompression, and stabilization (when required) of infection with a focus on neurological deficits and instability formation and non-stop severe pain are effective and safe treatment options.

## Introduction

With more than 500,000 new cases annually, human brucellosis is the most common zoonotic disease worldwide [1]. It is a systematic infection caused by facultative intracellular bacteria of the genus *Brucella *and can involve many organ systems. The musculoskeletal system is the most commonly involved in brucellosis capable of affecting different organs and tissues [2]. Although the prevalence of osteoarticular involvement changes depending on age and infecting type of *Brucella*, a rate of 5-85% has been reported in the literature [3,4]. Spinal involvement occurs in the adjacent disc together with the vertebra in 90% of the cases. First, vertebra corpus involvement is observed in spinal brucellosis, which then spreads to the adjacent disc space and adjacent vertebra corpus, with spondylitis turning into spondylitis spondylodiscitis. Most commonly, the lumbosacral region, especially L4-L5 vertebral involvement, is observed in spinal brucellosis [5]. Osteoarticular involvement may cause neurological and vascular complications [4,6].

Clinical characteristics of spinal brucellosis are non-specific and can be mistaken for other diseases [7]. Late onset of radiological findings and the complex nature of serodiagnosis also complicate the diagnosis of spinal brucellosis [8]. Although new guidelines have been suggested in the literature, its treatment remains controversial [9]. Frequent recurrences, treatment failure, and sequelae are constantly reported [10]. If spinal brucellosis is not treated appropriately, it may cause severe sequels, such as chronic lower back pain, neurological deficit, and even kyphotic deformity [5].

Antibiotics constitute the main treatment of spinal brucellosis and generally have a good prognosis. However, surgical treatment may be required in cases with neurological deficit, spinal instability formation, and severe pain irresponsive to surgical treatment. A limited number of studies are available in the literature on the surgical treatment of brucellosis-related spondylodiscitis. This study aimed to define and discuss the role of surgery in brucellosis-related spondylodiscitis.

## Materials and methods

A retrospective study was conducted among patients with spontaneous (non-postoperative) brucella spondylodiscitis. All patients were treated in the Department of Neurosurgery of a tertiary state hospital in Usak between February 2021 and August 2022. Usak Training and Research Hospital serves a community with a population of 750,000 together with neighboring settlements.

Patients who were diagnosed with brucella spondylodiscitis due to clinical, laboratory (biochemistry, microbiology), and radiological findings; provided written consent for the study; and did not have a history of spinal operation were included in the study. Patients who had a history of spinal surgery and/or neoplasm and were under 18 years of age were not included in the study.

Pre-diagnosis of brucella spondylodiscitis was based on the combination of clinical (localized pain, fever, neurological defect), laboratory (high C-reactive protein (CRP) and leukocytosis), and radiological findings (images of disc impairment in spinal CT and/or MRI compatible with spondylodiscitis). The diagnosis was confirmed through the isolation of *Brucella *species from blood and/or a standard agglutination test with a titer of ≥1/160 antibody titers to *Brucella*.

Surgical indications in our study included the following: (1) persistent pain due to spinal instability; (2) severe or progressive neurological deficit; spinal and nerve root compression by inflammatory granuloma and epidural abscesses; and (3) extensive lower back and leg pain non-responsive to antibiotic treatment. All surgeries were conducted by the same surgeon. Blood loss, total operation duration, and intraoperative complications were recorded.

Surgical techniques

Two types of surgical techniques were used in the patients, namely, (1) laminectomy, debridement decompression, and biopsy; and (2) laminectomy (decompression), debridement, posterior instrumentation (stabilization), and fusion. Patients were operated on in a prone position under general anesthesia in the first technique. Unilateral or bilateral hemilaminectomy was conducted depending on the condition of compressing from the spinal cord and nerve roots from the posterior. Epidural abscess, infected disc, and granulation tissues were debrided. Removed tissues and abscesses were sent for histopathological examination.

**Figure 1:**
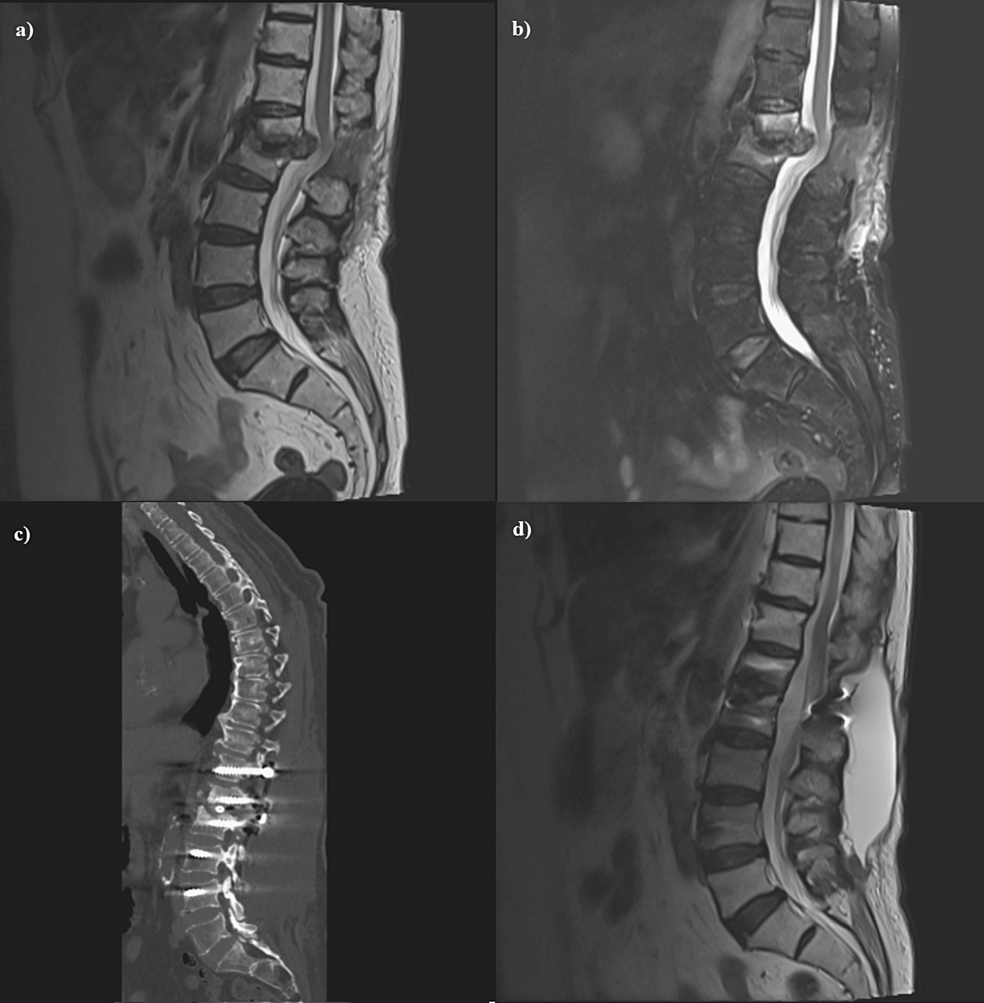
A 66-year-old female presenting with L1-L2 spinal brucellosis. (a and b) Sagittal T1 and T2-weighted MRI images showing lesions involving L1-L2 vertebral bodies and intervertebral discs. Epidural abscess and inflammatory granuloma formation. (c) Postoperative CT shows posterior interbody fusion and bilateral transpedicular titanium screws. (d) Six-month postoperative T2-weighted MRI images.

Patients were operated on in a prone position under general anesthesia in the second technique. Total laminectomy and discectomy were performed at the affected spinal level due to infection. Epidural abscess, infected disc, and granulation tissues were debrided. Removed tissues and abscesses were sent for histopathological examination. A polyether ether ketone cage was inserted for posterior interbody fusion in the discectomy distance. Subsequently, bilateral transpedicular titanium screws were inserted in affected vertebra distances under C-armed fluoroscopy (Figure 1).

Local antibiotic treatment with 0.75-1.75 g streptomycin was routinely applied to the surgical area. Drains were left on the wound for serum and antibiotic lavage for five to seven days postoperatively.

Patient analysis and follow-up

If the patients were completely pain-free in the follow-up (or if the pain had an intensity allowing normal daily activities) and if no radiological progression or spinal instability was detected in the last control, the patients were considered recovered. We know that the MRI findings of spondylodiscitis do not clearly improve until several months after surgical debridement. The following criteria were used in CT/MRI for radiological recovery: radiological stability of the segment, proper positioning of the implanted material, and absence of displacement or breakage in material and segment vertebral fusion. Patients who presented with permanent neurological deficits or pain requiring analgesics at the end of follow-up were considered sequelae.

Intravenous antibiotics were routinely administered for three days postoperatively. All patients received the World Health Organization-suggested oral treatment including 200 mg doxycycline and 600-900 mg rifampicin for a minimum period of three months in the postoperative period. All patients were mobilized by a lumbosacral brace on the third day postoperatively. CT imaging was conducted to evaluate the location of instrumentation before discharge in patients using instrumentation.

Patients were followed up after one, three, and six months. Medical records, CT/MRI imaging findings, laboratory values, and neurological and functional conditions were recorded and analyzed. Using CT scans, fusion was evaluated using the Bridwell criteria. The erythrocyte sedimentation rate (ESR) and C-reactive protein (CRP) values of the patients were analyzed. The Visual Analog Scale (VAS) was used to evaluate lower back pain. Pain-related dysfunction was evaluated using Oswestry Disability Index (ODI).

Statistical analysis

Data analysis was done using SPSS version 20 (IBM Corp., Armonk, NY, USA) program. Student’s t-test was used to evaluate preoperative and final follow-up changes in the laboratory (ESR, CRP) and quantitative scores (VAS, ODI). P-values less than 0.05 were considered significant.

## Results

A total of 28 (16 males, 12 females) patients who underwent surgical treatment due to brucellosis-related spondylodiscitis were included in this study. The mean age of the patients was 56.4 ± 7.2 years, and the mean follow-up duration was 11.8 ± 5.4 months. All patients had spinal pain in line with the affected vertebra level. Brucella spondylitis involvement was found at the L4-L5 level in 10 patients, at the L5-S1 level in seven patients, at the L2-L3 level in six patients, at the L3-L4 level in four patients, and at the L1-L2 level in one patient. Sixteen (57.1%) patients had radicular symptoms (leg pain, hypoesthesia) (Table 1).

**Table 1:** Clinical and epidemiological characteristics of brucella spondylodiscitis.

	Number of patients (%)
Sex	
Female	12 (42.9%)
Male	16 (57.1%)
Median age (years)	56.4 ± 7.2
Spinal symptoms	
Back pain	28 (100%)
Radiculopathy	16 (57.1%)
Neurological defect	
Motor	4 (14.3%)
Motor + sensory	17 (60.7%)
Surgical treatment	
Laminectomy + debridement	8 (28.6%)
Posterior stabilization + fusion	20 (71.4%)
Operation duration (minute)	112.4 ± 28.6
Blood loss (mL)	238.3 ± 91.9

In total, 21 patients had neurological deficits in the preoperative period. Brucellosis involvement was in the lumbosacral region in all patients. Surgery was successful in all patients. Posterior stabilization and fusion were applied in 20 (71.4%) patients, while simple laminectomy (decompression) and debridement were applied in eight (28.6%) patients. The average operation duration was 112.4 ± 28.6 minutes, and the average blood loss was 238.3 ± 91.9 mL. *Brucella *agglutination test was ≥1/160 in all patients, and blood culture was positive in 14 (50%) cases. Non-caseating granulomatous inflammation was detected in all histopathological examinations. There was a decrease in pain in the lower back and leg of all patients in the postoperative period. Neurological recovery was achieved in 18 patients with neurological deficits. Debridement was required in the postoperative period in two patients due to wound drainage and infection. Both of these patients had diabetes and recovered following wound suturing and antibiotic treatment. Except for these two patients, there were no patients with recurrent infections in our follow-up. The patient who had morbid obesity and comorbidity died in the postoperative period. Twenty-four (85.7%) patients recovered at the end of treatment and follow-ups, three patients were regarded as sequelae, and one patient died. The average hospitalization duration was 13.4 days. According to Bridwell criteria, grade I fusion was seen in 26 patients, and grade II fusion was noted in the remaining two patients. The total rate of fusion was 92.9%.

ESR and CRP levels returned to normal at the end of the six-month follow-up. Mean ESR and CRP levels of 32.51 ± 9.93 and 44.32 ± 16.82 in the preoperative period reduced to 2.52 ± 1.24 and 3.32 ± 2.03, respectively, at the sixth-month follow-up (Table 2).

**Table 2:** Mean ESR and CRP levels. ESR = erythrocyte sedimentation rate; CRP = C-reactive protein

Parameters	Preoperative	Follow-up	P-value
ESR	32.51 ± 9.93	2.52 ± 1.24	<0.05
CRP	44.32 ± 16.82	3.32 ± 2.03	<0.05

There was a significant statistical recovery in VAS and ODI values (p < 0.05) (Table 3).

**Table 3:** Clinical and epidemiological characteristics of brucella spondylodiscitis. VAS = Visual Analog Scale; ODI = Oswestry Disability Index

Parameters	Preoperative	Follow-up	Improvement rate (%)	P-value
VAS	8.43 ± 3.18	1.10 ± 0.41	86.9	<0.05
ODI	48.42 ± 7.96	11.25 ± 4.12	76.8	<0.05

## Discussion

The incidence of brucella spondylodiscitis had a rapid global increase, especially in underdeveloped regions, in recent years [11]. Despite its high prevalence and effective antibiotic treatment, the diagnosis and treatment of the disease remain difficult [12]. Late onset of radiological findings and the complex nature of serodiagnosis complicate the diagnosis of spinal brucellosis. Most spinal brucellosis patients can be treated without operation. Koubaa et al. reported 32 spinal brucellosis cases in which complete recovery was achieved both radiologically and clinically through antibiotics-only treatment [13].

Due to late diagnosis and treatment, neurological deficit, spinal instability-related persistent or progressive lower back pain, and large paravertebral abscess formation may occur in some spondylodiscitis cases [14,15]. Some cases may not respond to antibiotic treatment. Surgical treatment is performed in such patients [16,17]. In our study, patients who had spinal instability, severe or progressive neurological deficits, and did not respond to antibiotics were diagnosed with surgical treatment indications. Surgical treatment of brucella spondylodiscitis has been reported rarely in the literature, and surgical intervention has a controversial role.

Biochemical markers and laboratory tests, such as ESR, CRP, and leukocyte counts (WBC), are important in the follow-up of patients treated due to spondylodiscitis. Carrage et al. reported that initial ESR values below 25% were a good prognosis marker and that CRP returned to the normal range in approximately three months [18]. The results of our study are in line with the literature. ESR and CRP levels returned to normal at the end of the six-month follow-ups in all patients.

Tschöke et al. stated that moderate wide debridement together with the antimicrobial treatment provided recovery in patients with spondylodiscitis [19]. Although open and wide surgical techniques have been suggested in the literature, minimally invasive or endoscopic techniques for spondylodiscitis have started to stand out recently. Minimally invasive surgery, bilateral decompression, and microdiscectomy through a one-sided approach) were done in three patients with obesity and severe comorbidities in our study. This approach facilitated medullar compression in only one session. While a complete radiological and clinical recovery was achieved in two patients in whom we used this technique, one patient died due to non-treatment-related reasons. We think that this technique can be evaluated better in future studies with a higher number of cases.

Spinal brucellosis typically emerges in the lumbosacral region, especially at the L4-L5 levels [3,5]. Brucellosis involvement was in the lumbosacral region in all patients in our study. Different surgical techniques, including anterior, posterior, and combined approaches, have been described for spinal infection surgery [6]. Anterior approaches are not enough to provide decompression of the contralateral nerve root [6]. Spinal brucellosis is less destructive compared to other infectious spinal diseases, and thus minimally invasive methods should be preferred as much as possible in surgical intervention. A screw can be inserted in the affected corpus in patients where stabilization is planned due to patients presenting without severe corpus damage in spinal brucellosis, and an operation can be planned as short-segment instrumentation. Laminectomy (decompression) was applied in all patients without instability finding (n = 8) in our study. Posterior stabilization and fusion were applied in all patients with instability (n = 20). Lee et al. reported good results for posterior lumbar interbody fusion and posterior instrumentation in lumbar tuberculosis surgery [20]. Thirty-two patients operated on in the lumbosacral region due to spinal brucellosis were evaluated in a study by Abulizi et al. Posterior instrumentation and fusion were applied in all patients [6]. It was reported that full recovery was seen in all patients in the postoperative period, and reoperation was not required. In a study by Bydon et al. including a high number of cases, patients who underwent only decompression and 118 spinal infection patients who underwent instrumentation were compared, and no difference was reported in reoperation and superinfection rates [21]. Only two patients in our study (one patient from the laminectomy group and one patient from the posterior stabilization and fusion group) had wound drainage. Both patients were obese and had diabetes. These patients recovered through wound suturing and antibiotic treatment. No instrumentation inadequacy or instability was recognized during patient follow-ups. Reoperation was not needed in any of our patients. Complete radiological and clinical recovery was achieved in 24 (85.7%) patients. Neurological recovery was achieved in 85.7% of the patients with neurological deficits. All patients were operated on within 24 hours following surgical treatment indication. It was observed that the pain mostly regressed in all patients in the postoperative early period, and they were mobilized in the early period. The follow-ups of patients showed that surgical treatment (with or without instrumentation) fastened recovery and infection eradication, preserved spinal mechanics, and did not cause superinfections. No intraoperative complication was observed in our cases, and back and leg pain was observed to decrease significantly in all patients in the postoperative period. Recovery rates in VAS and ODI scores were 86.9% and 76.8%, respectively. Recurrence was not detected in any patient during follow-up.

We think that the operation without losing time, provision of neuro and medullary decompression, and performance of wide debridement in patients who decided to have surgical treatment were effective in decreasing pain complaints of patients in the early period, high neurological recovery, and quick response to medical treatment. We also think that the surgical applications performed in only one session increased treatment success even in elderly patients with comorbidities.

Due to the low number of patients who underwent only laminectomy in our study, no comparison was possible between patients who underwent only laminectomy and those who underwent instrumentation in terms of blood loss during operation, total operation duration, complication rate, and recovery rate. This was a limitation of this study.

## Conclusions

Brucellosis-related spondylodiscitis, or brucellosis, is a disorder of the body’s nervous and circulatory systems that leads to neurological deficits, instability formation, and non-stop severe pain. Although long-term and specific antibiotic treatment is the main treatment modality, debridement and decompression can be effective and safe treatment methods. Surgeries conducted without losing time fasten the recovery phase and mobilization of patients. Surgical treatment does not increase the risk of superinfections in patients. After spinal brucellosis surgery was instrumented, positive outcomes were found. High neurologic recovery rate and fusion rate were found in patients. There was a significant reduction in the pain of the patients. Therefore, when necessary, the use of instruments in spinal brucellosis surgery should not be avoided.
